# Health-related quality of life of refugees: a systematic review of studies using the WHOQOL-Bref instrument in general and clinical refugee populations in the community setting

**DOI:** 10.1186/s13031-021-00378-1

**Published:** 2021-06-02

**Authors:** Juliette Gagliardi, Christian Brettschneider, Hans-Helmut König

**Affiliations:** 1 Department of Health Economics and Health Services Research, Hamburg Center for Health Economics, University Medical Center Hamburg-Eppendorf, Martinistrasse 52, 20246 Hamburg, Germany; 2 Department of Economics, University of Bologna, Via Zamboni 33, Bologna, 40126 Italy

**Keywords:** Health-related quality of life, WHOQOL-Bref, Refugee, Trauma, Mental health disorders

## Abstract

**Purpose:**

To systematically review studies on HRQOL, measured by the WHOQOL-Bref instrument, of refugees in general and clinical populations who are settled in the community of the hosting country, and outline the differences in scores among the two population groups and across the four domains of WHOQOL-Bref (physical, psychological, social relationships and environment domain) as well as factors impacting those outcomes.

**Methods:**

Several databases were systematically searched by using a broad search strategy. Additionally, a hand search for grey literature was performed. Studies had to comply with the following inclusion criteria: (a) population of refugees; (b) living in the community of the country of destination; (c) assessing HRQOL through the WHOQOL-Bref instrument.

**Results:**

15 studies were identified and divided into two subgroups: (a) general population of refugees (b) clinical population of refugees, who were specifically selected for their mental status or because they had experienced relevant past traumas. Although we can outline common patterns among the two groups, in terms of domains scoring the highest and the lowest, heterogeneous values of HRQOL are observed across the studies included.

**Conclusions:**

Individuals who were included in the clinical refugee group have a lower quality of life in respect to the general population of refugees. However, among the two groups different patterns can be outlined considering each domain of HRQOL: higher scores for the Physical and lower for the Environment domain when considering the general population of refugees and higher scores for the Environment and lower for the Psychological domain when referring to the clinical one. These lower scores are probably due to having a higher rate of mental distress and being more exposed to somatization, stigmatization and barriers to access the healthcare system of the hosting country.

**Supplementary Information:**

The online version contains supplementary material available at 10.1186/s13031-021-00378-1.

## Introduction

There are currently over 79.5 million people who are forcibly displaced in the world, among them 26 million are refugees, a number that more than doubled in the last decade [1]. Open-ended civil wars in Syria, Libya, Afghanistan, Iraq and South Sudan, severe economic and political instability in Venezuela, the persecution of Rohingya’s ethnic group and many other current conflicts, have been relevant in increasing the global migration wave in recent years [2]. Moreover, the impact of climate change has become also a primary driver for the increase of migration flows in particular when combined with economic stagnation [3]. Those migration flows affect the destination countries in relation to the features of the migrants and the characteristics of the countries themselves.

The large majority of refugees are exposed to traumas. In their country of origin, they experience pre-migration traumas like human right violations, murder of relatives, imprisonment, torture and war. In transit to their destination are often exposed to physical and sexual violence, as well as maltreatment by traffickers and authorities. Finally, after arrival at their destination, refugees are exposed to post-migration stressors, such as feelings of being alone and uprooted, social exclusion sometimes culminating into open hostilities, and hardships in securing their subsistence. Factors that put them under immense pressure and worsen an already precarious mental health state in refugees who have been already subjected to trauma. All these aspects are strongly associated with mental health problems, namely PTSD, depression and anxiety [4–16]. The impact is impressively illustrated by the fact, that the prevalence of PTSD, depression and anxiety among refugees is twice as high as the prevalence among labour migrants, which is 20% [17]. Although there is a wide literature in support of the positive effects that migration inflows have in the hosting countries economies, native populations and demographics [18–27], this specific group of migrants deserves peculiar attention, as having distinct needs and vulnerabilities, when it comes to integration which is a key element for migrants to contribute to the economic and civil life of their hosting countries. Indeed, health, with work and education, is a key indicator for social integration and inclusion.

As the experiences made by refugees might affect different aspects of health, it is necessary to employ a more general definition of health to describe the health status of this population. The health-related quality of life (HRQOL) is an outcome which aims to respond to the necessity of assessing health through a non-disease specific instrument «capturing changes of health that matter to the patients and the societies they live in» [28]. In this review, we chose this broad outcome to understand refugees’ health in the community setting and how their health status affects their lives in this context. Furthermore, we make a distinction between the general population of refugees and the subgroup of refugees with mental health problems of clinical significance. The first group represents the general health status of refugees. The other group gives insights into the connection between specific traumatic backgrounds and health status.

The holistic definition of health provided by the WHO (viz.“health is a state of complete physical, mental, and social well-being and not merely the absence of disease or infirmity” [29]) was at the basis of the creation of the WHOQOL-Bref, which is the most frequently used instrument to assess HRQOL among refugees as, due to its multiple versions adapted to different languages and cultural backgrounds, it allows direct comparisons between studies and covers multiple dimensions in a culturally adapted way [30–32]. This cultural adaptation is important, as personal perception led by cultural background has a great impact on the value of HRQOL [33].

In particular, the purpose of this paper was as follows:

- to systematically review studies examining HRQOL of refugees using the WHOQOL-Bref, considering both non-clinical and clinical groups, when living in a community setting;
- to identify factors that impact HRQOL;
- to provide a starting point for future investigations of individuals having refugee status, belonging to a general or clinical population;
- to inform possible policies aimed to enhance the HRQOL of refugees.

## Methods

### Search strategy

We conducted a systematic literature search in Medline, PubPsych, BioMed Central, CINAHL, APA PsychInfo, APA Psycarticles, Index Islamicus, Cochrane Library and the Open System for information on Grey Literature in Europe (OpenSIGLE). Furthermore, we scanned the references of the studies selected and conducted an additional hand search focusing on potentially relevant journals (e.g. the Journal of Refugee Studies). We chose a broad search strategy using the following terms: “refugee*”, “asylum seeker*”, “undocumented”, “Migrant*” and “Immigration” (due to possible inconsistencies in the definition of refugees) combined with “HRQOL”, “Health-related quality of life, “QOL” and “Quality of life”.

### Inclusion criteria

Studies meeting the following criteria were included: (a) population of refugees; (b) living in the community of the country of destination and being resettled in High-Income Countries (HICs) or in Upper-Middle Income Countries (UMICs) as classified by the World Bank criteria [34]; (c) assessing HRQOL through the WHOQOL-Bref evaluation tool. No restriction was set regarding the publication type. However, in case of intervention studies just the cross-sectional baseline data were taken into consideration to enable comparison with the other studies. No limits were tied to the language and the date of publication.

The eligibility check consisted of two steps. First titles and abstracts were checked for potentially relevant content. Second, publications deemed potentially relevant were screened in full text.

### Type of participants

We considered refugees as defined by the Protocol of the Refugee Convention in 1951 as any person “who is unable or unwilling to return to their country of origin owing to a well-founded fear of being persecuted for reasons of race, religion, nationality, membership of a particular social group, or political opinion” [35]. In the studies selected, we aimed to consider specifically a population having obtained the status of refugee. We excluded unapproved asylum seekers and undocumented as they might be subjected to a different perspective for a new life and integration process in the host country, due to the impossibility to work legally, a constant fear of being repatriated or lack of full access to the healthcare system. Those elements might influence their HRQOL making their outcome not comparable with the population of interest [36–38].

### Setting

It appeared to be appropriate to observe a more homogeneous population living in comparable environments, facing the same opportunity and difficulties of being integrated into a new country. Therefore, we decided to exclude those studies taking into consideration individuals living in a more precarious situation, as refugee camps, specific housing facilities or detention centres, which often represents a phase of transition towards a new dwelling and where it is observed a higher rate of depression [13] and abuse of substances [39].

### Diagnosis

We made a distinction between refugees belonging to the general population group of refugees and a clinical one. Among the first group, the selection was based solely on the participants refugee status. The second group of refugees was selected among people having experienced highly traumatizing events or having a formal diagnosis of PTSD, anxiety and/or depression or scoring above the cutoff values using self-report instruments such as Hopkins Symptom Check List-25 (HSCL-25), for anxiety and depression, and Harvard Trauma Questionnaire-PTSD (HTQ-PTSD) for diagnosis of post-traumatic stress disorder. It has been observed that refugees having been exposed to comparable types of stress produce different symptomatic patterns [40] and that cultural and societal values play a fundamental role in determining how mental health symptoms manifest [41–43]. Therefore, it is relevant to outline the effects that those episodes have on the mental state of the ones who undergo those adversities, using culturally sensitive instruments as the ones above mentioned.

### Outcomes measure

The WHOQOL-Bref consists of a 24-item self-reported questionnaire divided into four domains (physical, psychological, social relationships, and environmental) plus 2 items to measure individual perceptions of global QOL and health status [44, 45]. Outcomes with this tool can be shown using different scales: the raw one and the ones having a range from 0 to 20 and from 0 to 100. When needed, we converted all the results using a single scale to make the comparisons more straight forward [46]. This conversion does not affect the outcome itself as it just shows the results in a different range. We opted for the scale 0–100, where 0 stands for extremely poor and 100 for exceptionally good QoL. 50 can be considered the cutoff score at which the outcome is neither good nor poor [47]. The WHOQOL-Bref was conceived to be used across different cultures and was shown to be effective in the comparison of subgroups within the same culture [47, 48].

### Data extraction

The data extraction was designed to show the main characteristics of the studies and the samples taken into consideration, as well as their primary outcomes. As baseline characteristics we extracted: sample size, gender, age mean and marital status of the participant, their country of origin and the country where the study was performed. Information regarding the characteristics of the study outlined in the table are: study location, recruitment method and year of data collection. When considering the general population of refugees only the values of WHOQOL-Bref was evaluated as an outcome.

From studies considering the population of refugees with clinically relevant mental health problems we extracted also their employment status and duration of stay in years, and outcomes evaluating the psychological status of the participant in terms of anxiety, depression and PTSD. Regarding those outcomes, we extracted information on the average scores of the questionnaires used to evaluate their mental health condition and, in some cases, the percentage of the sample suffering from those mental disorders. Information that was not displayed consistently among studies or in a non-comparative form, such as education level, were excluded.

### Quality assessment

The well-known and widely used NIH Quality Assessment Tool for Observational Cohort and Cross-Sectional Studies [49] was used to assess the study quality.

## Results

### Selected studies

**Fig. 1:**
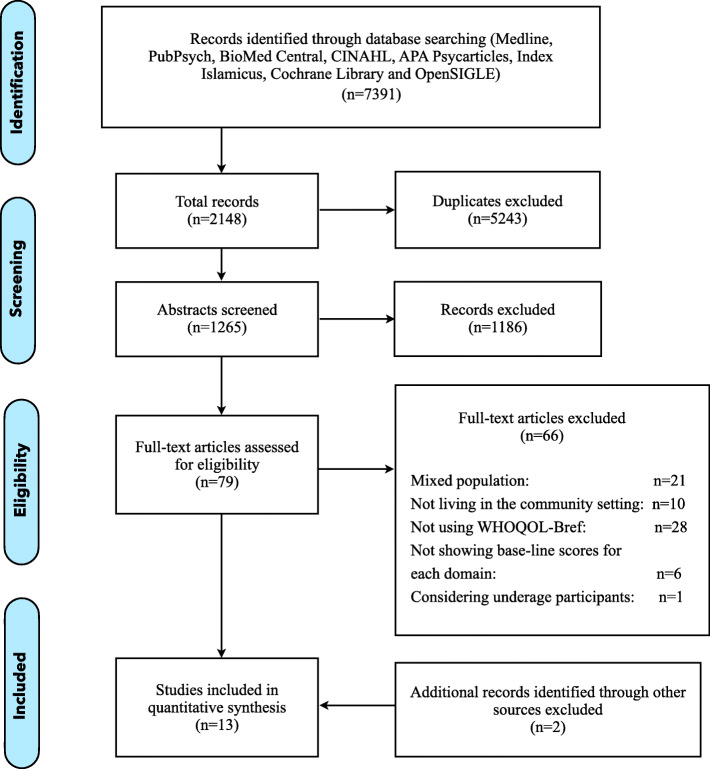
PRISMA flow diagram

The initial search generated 7391 results. 5243 were duplicates. After abstract screening, 79 publications were deemed potentially relevant. 66 studies were excluded as they were not meeting the inclusion criteria. 21 were excluded because considering a mixed population of refugees and asylum seekers, or not clearly defining their status; 10 were considering refugees living in camps, housing facilities or detention centers; 28 were not using the WHOQOL-Bref but other instruments to assess the HRQOL (SF-36, SF-12 and WHO-5 were the ones used more frequently); 6 were excluded because they were not displaying the outcomes of WHOQOL-Bref at the baseline making those incomparable to the other studies included; 1 study considered underage participants which are characterized by other vulnerabilities and needs in respect to an adult population. Additional screening of reference lists and additional hand search considering relevant journals, resulted in identifying two further publications. Among the studies selected, 11/15 were performed in High-Income Countries and none of them were RCTs. Detailed information is provided in the PRISMA flow diagram in Fig.1. Sample sizes ranged from 22 to 655, with a total sample of 2352 across studies (1533 included in the general population of refugees and 819 in the clinical one), of which 56.5% males (46.4% considering just the general population and 75.6% in the clinical one).

### General population of refugees

All studies in this group were observational studies. The aim of these studies was diverse: to compare differences between refugees and local population [50] or between life in a refugee camp and outside [51, 52] (in this last case observing higher scores); to assess the HRQOL in a determined country of arrival [50–55] and often considering refugees coming from the same country [50, 51, 53, 55], specifically Syria, Palestine and Somalia.

Table 1 shows characteristics of the studies selected. We observe a large heterogeneity in the scores of HRQOL across the studies included: from a minimum score of 38.0 for the environmental domain, to a score of 73.1 for the physical domain. Those two domains are also the ones scoring the lowest and the highest within all studies, except for the study Redko et al. [55] where the lowest score was observed for the physical domain and the highest in the psychological one. In Crea et al. [52] the psychological domain and social relationship one were not assessed because the authors believed that indicators such as spirituality, body image and self-esteem for the first domain, and sexual activity and personal relationships for the second, would not be understood or would have been perceived as a violation of privacy by the sub-Saharan African individuals participating to the study.

**Table 1:** Study and socio-demographic characteristics, WHOQOL-Bref scores of respondents belonging to the general population of refugees

	Abdo et al. (2019) [50]	Alduraidi et al. (2017) [51]	Crea et al. (2015) [52]	Georgiadou et al. (2020) [53]	Horta et al. (2019) [54]	Redko et al. (2015) [55]
**Study characteristics**						
Study location	Al-Husun, Jordan	Abu Nsair, Jordan	Johannesburg and Pretoria, South Africa	Erlangen, Germany	Brazil	Columbus, Ohio (USA)
Recruiting method	registry-based	field-based	registry-based	registry-based	snowball sampling	field-based
Year of data collection	Feb-Aug 2017	Oct- Nov 2015	June 2012- Aug 2013	July- Dec 2017	Aug 2016- April 2018	Sept 2012- June 2013
**SAMPLE characteristics**						
Size	655	91	334	119	31	303
Gender (n, %)						
Male	266 (40.6%)	44 (48.4%)	170 (50.9%)	71 (59.7%)	3 (9.7%)	157 (51.8%)
Female	389 (59.4%)	47 (51.6%)	149 (44.9%) (a)	48 (40.3%)	28 (90.3%)	146 (48.2%)
Age mean (SD)	–	36.0 (14,4)	–	38.82 (−)	–	46.5 (−)
Marital status (n, %)						
Married	586 (89.4%)	60 (65.9%)	–	119 (100%)	–	164 (54.1%) (c)
Non married (b)	69 (10.6%)	31 (34.1%)	–	0	–	129 (42.6%)
Country of origin	Syrian	Palestine	Diverse	Syrian	Diverse from Africa	Somalia
Country of destination	Jordan	Jordan	South Africa	Germany	Brazil	USA
**WHOQOL-Bref mean (SD)**						
Physical health	50.68 (−)	64.4 (18.0)	58.9 (17.5)	73.10 (−)	56.9 (−)	44.69 (18.30)
Psychological health	49.35 (−)	56.5 (19.5)	–	65.39 (−)	52.9 (−)	52.83 (18.44)
Social relationships	49.82 (−)	58.3 (21.5)	–	68.38 (−)	56.3 (−)	50.04 (20.36)
Environment domain	47.37 (−)	51.3 (15.3)	38.0 (14.9)	60.45 (−)	38.0 (−)	46.32 (16.87)

WHOQOL-Bref, World Health Organization Quality of Life Assessment in its short version, scaled 1–100, with domains for physical health, psychological health, social relationship and environmental domains. For each domain the mean score of WHOQOL-Bref is reported and, when available, the standard error

(a) 15 (4.2%) missing information regarding gender

(b) “non-married” comprises single, divorced and widowed

(c) 10 respondents didn’t provide information regarding their marital status

Most studies showed no clear gender differences and no evident distinction between studies performed in HICs and UMICs can be observed.

### Clinical population of refugees

Table 2 outlines the baseline characteristics of the studies and population samples selected for the group of the clinical population of refugees. We can distinguish two kinds of studies in this group: 1) epidemiological studies considering a group of refugees, who are characterized by a particular history of trauma [56–59]; 2) interventional studies evaluating the effectiveness of a certain treatment [60–64]. Five of these studies took into consideration specifically refugees who had experienced torture [56, 60–63] a factor that has been shown to be substantially associated with PTSD [13].

**Table 2:** Study and socio-demographic characteristics, mental health disorders scores and WHOQOL-Bref scores of respondents belonging to the clinical group of refugees

	Carlsson, Mortensen et al. (2006) [56]	Huijts et al. (2012) [57]	Opaas & Varvin (2015) [58]	Teodorescu et al. (2012) [59]	Carlsson et al. (2005) [60]	Carlsson et al. (2010) [61]	Carlsson, Olsen et al. (2006) [62]	Kinzie et al. (2012) [63]	Opaas et al. (2016) [64]
**Study characteristics**									
Study location	Copenhagen, Rehabilitation and Research Centre for Torture Victims (RCT), Denmark	Foundation Centrum ‘45, Oegstgeest, The Netherlands	Two general mental health outpatient clinics and six psychotherapists in publicly funded individual practices, Oslo (Norway)	Outpatient departments of four hospitals from South- Eastern Norway	Copenhagen, Rehabilitation and Research Centre for Torture Victims (RCT), Denmark	Copenhagen, Rehabilitation and Research Centre for Torture Victims (RCT), Denmark	Copenhagen, Rehabilitation and Research Centre for Torture Victims (RCT), Denmark	Refugee clinic, Oregon (USA)	Specialist mental health outpatient services with public funding, Norway
Recruiting method	direct recruitment method	direct recruitment method	direct recruitment method	direct recruitment method	direct recruitment method	direct recruitment method	direct recruitment method	direct recruitment method	direct recruitment method
Year of data collection	1 Jan 2001–15 May 2002	2003–2010	2006–2009	1 Nov 2008- Nov 2009	2001–2002	1 Jan 2001–15 May 2002	1991–1994	Feb 2009- Jan 2010	2006–2009
**SAMPLE characteristics**									
Size	63	335	54	55	55	45	139	22	51–49
Gender (n, %)									
Male	63 (100%)	251 (74.9%)	35 (64.8%)	32 (58%)	40 (72.7%)	30 (66.7%)	126 (90.6%)	9 (41%)	33 (64.7%)
Female	–	84 (25.1%)	19 (35.2%)	23 (42%)	15 (27.3%)	15 (33.3%)	13 (9.4%)	13 (59%)	18 (35.3%)
Age mean (SD)	37.8 (8.11)	41.9	39.3 (8.2)	42 (8.6)	–	39.2 (9.38)	44.7 (8.48)	48 (−)	39.4 (8.0)
Marital status (n, %)									
Married	50 (79.4%)	–	35 (64.8%)	32 (58.2%)	43 (78.2%)	37 (82.2%)	87 (62.6%)	–	34 (66.7%)
Non married (a)	13 (20.6%)	–	19 (35.2%)	23 (41.8%)	12 (21.8%)	8 (17.8%)	52 (37.4%)	–	17 (33.3%)
Country of origin	Diverse	Diverse	Diverse	Diverse	Diverse	Diverse	Diverse	Diverse	Diverse
Country of destination	Denmark	The Netherlands	Norway	Norway	Denmark	Denmark	Denmark	USA	Norway
Duration of stay in years	–	12.8 (4.73)	10.5 (6.5)	16.7 (7.1)	–	5.8 (4.98)	4.9 (−)	–	11.2 (6.3)
Unemployed	–	–	42 (77.8%)	31 (59.6%)	–	–	103 (73.9%) after 10 years	majority	40 (78.4%)
HSCL-25									
ANXIETY mean (SD)	2.90 (0.67)	–	2.89 (0.59)	–	2.84	2.82 (0.73)	2.26 (0.74)	–	2.87 (0.59)
Above cut-off	91.9%	–	96.2%	94.5% (x)	87.3%	–	74.1%	–	96%
DEPRESSION mean (SD)	2.91 (0.63)	–	2.94 (0.54)	2.7 (0.64)	2.88	2.81 (0.67)	2.25 (0.70)	100% (Center for Epidemiological Studies—Depression Scale)	2.94 (0.52)
Above cut-off	93.5%	–	98.1%	93%	92.7%	–	69.1%		98%
HTQ - PTSD									
Mean (SD)	3.06 (0.53)	2.91 (0.57)	2.82 (0.47)	–	2.93	2.91 (0.60)	2.61 (0.68)	–	2.82 (0.47)
Above the cut-off	88.9%	85%	78,8%	80% (IESR)	78.2%	–	56.1%	77.3% (SPRINT)	81.6%
**HRQOL mean(SD)**									
Physical health	25.57 (20.03)	38 (−)	28.5 (13.8)	32.5 (14.7)	29.55 (−)	30.19 (20.32)	46.28 (21.65)	31 (−)	28.2 (13.8)
Psychological health	27.52 (19.87)	44 (−)	25.6 (15.9)	34.6 (15.5)	31.04 (−)	32.94 (19.84)	42.70 (22.55)	31 (−)	25.3 (15.7)
Social relationships	39.66 (25.12)	44 (−)	36.6 (23.4)	43.4 (19.7)	41.59 (−)	42.13 (23.84)	46.55 (24.61)	–	35.0 (21.7)
Environment domain	39.87 (19.00)	50 (−)	45.2 (18.2)	43.1 (15.1)	39.03 (−)	37.78 (17.81)	49.44 (19.53)	47 (−)	45.0 (18.5)

WHOQOL-Bref, World Health Organization Quality of Life Assessment in its short version, scaled 1–100, with domains for physical health, psychological health, environmental and social relationship. Mean scores are Hopkins Symptoms Checklist-25 (HSCL-25) to measure anxiety (items 1–10) and depression (items 11–25), cutoff score 1.75; Harvard Trauma Questionnaire (HTQ) part IV (items 1–16) to measure PTSD, cutoff score 2.5. For HSCL-25 and HTQ mean scores and percentage of individuals scoring above the cutoff are outlined, for the WHOQOL-Bref just the mean score and, when available, the standard error

Unemployment was not reported when for “employed” the authors considered the participant taking part in language courses, job training, education and having an actual job

Education was not reported due to the heterogeneity on how education levels were reported

If the mental status has not been assessed with the HTQ or the HSCL-25, in parenthesis it is outlined the name of the other evaluation tool used for the purpose or “x” in case it was not specified

(a) “non-married” comprises single, divorced and widowed

The majority of the studies were performed in specialized psychiatric and rehabilitation facilities located in HICs. The samples included individuals who were recruited through a direct recruitment method. The Harvard Trauma Questionnaire was the instrument most frequently used to diagnose PTSD. The cutoff score considered was ≥2.5 for all the included studies, which is the standard cutoff for this instrument [65]. The Hopkins Symptom Checklist-25 was the most used tool to assess symptoms for anxiety and depression. It consists of two subscales, a 10-item anxiety scale and a 15-item depression scale, and the score > 1.75 is considered positive for major depressive disorder and clinical level of anxiety. Evidence for the validity and reliability of the HTQ and the HSCL-25 across different groups of refugees with a variety of linguistic backgrounds has been shown by a variety of studies [66–71].

In epidemiological studies, the researchers aimed to observe an association between different elements characterizing this population and their HRQOL. We want to highlight the following findings: in the study of Teodorescu et al. [59], depressive and posttraumatic stress symptoms were negatively associated with all domains of HRQOL, while moderate to large significant positive correlations were found in regard to posttraumatic growth. In the study of Opaas & Varvin [58], decreased HRQOL, particularly low in the psychological domain, was the result of repeated traumatic childhood events, e.g.intra and extra-familial violence. 91% of the participants of this study made these experiences.

We do not observe peculiar differences in HRQOL’ outcomes at the baseline, comparing intervention and epidemiological studies. The only scores, which are noteworthy by being above the outcomes reported by the epidemiological studies, are the ones of Carlsson, Olsen et al. [62]. Here, we also observe a lower percentage of participants diagnosed with anxiety, depression and/or PTSD.

On average, the scores of WHOQOL-Bref for the group of clinical refugees are lower in comparison to the ones of the general population of refugees but we can still notice some exceptions, especially when looking at the environmental domain. The lowest outcomes were observed for the physical and the psychological domain, for which scores below 30.0 were assessed, while the highest were observed for the environmental domain reaching a value of 50.0 in the sample considered by Huijts et al. [57]. These lower outcomes can be explained by the a priori selection that was done for these studies, considering highly traumatized and highly symptomatic refugees. Indeed, it has been found that factors such as depression [72], anxiety and PTSD [73] lead to a lower HRQOL.

### Quality assessment of included studies

Table 3 provides in detail the quality assessment of the included studies which has been done using the Quality Assessment Tool for Observational Cohort and Cross-Sectional Studies provided by the National Institute of Health (NIH). We can notice that for all studies included it was important to clearly specify and define the population of interest. The subjects were recruited from same or similar populations and no exceptions were done when applying exclusion criteria during the selection process. However, for almost all the studies, risk of bias was mainly due to a lack of sample size justification, that appeared to be not truly representative of the population. For some of them we observe a lack of consistency regarding how the questionnaire was administered across participants (self-administered, using professional interviewers and with the translation of interpreters when needed). Overall, four studies were rated as good quality, nine as fair and two as Poor. Further characteristics of the included studies can be found in the Supplementary file 1.

**Table 3:** Quality Assessment Tool for Observational Cohort and Cross-Sectional Studies provided by the NIH

	1	2	3	4	5	6	7	8	9	10	11	12	13	14	15
1. Was the research question or objective in this paper clearly stated?	**Y**	**Y**	**Y**	**Y**	**Y**	**Y**	**Y**	**Y**	**Y**	**Y**	**Y**	**Y**	**Y**	**Y**	**Y**
2. Was the study population clearly specified and defined?	**Y**	**Y**	**Y**	**Y**	**Y**	**Y**	**Y**	**Y**	**Y**	**Y**	**Y**	**Y**	**Y**	**Y**	**Y**
3. Was the participation rate of eligible persons at least 50%?	***CD***	**Y**	**Y**	**N**	***NR***	***CD***	**Y**	***NR***	**Y**	**Y**	**Y**	**Y**	**Y**	**Y**	**Y**
4. Were all the subjects selected or recruited from the same or similar populations (including the same time period)? Were inclusion and exclusion criteria for being in the study prespecified and applied uniformly to all participants?	**Y**	**Y**	**Y**	**Y**	**Y**	**Y**	**Y**	**Y**	**Y**	**Y**	**Y**	**Y**	**Y**	**Y**	**Y**
5. Was a sample size justification, power description, or variance and effect estimates provided?	**Y**	**Y**	**N**	**N**	**N**	**N**	**N**	**N**	**N**	**N**	**N**	**N**	**N**	**N**	**N**
6. For the analyses in this paper, were the exposure(s) of interest measured prior to the outcome(s) being measured?	***NA***	***NA***	***NA***	***NA***	***NA***	***NA***	***NA***	***NA***	***NA***	***NA***	**Y**	**Y**	**Y**	**Y**	**Y**
7. Was the timeframe sufficient so that one could reasonably expect to see an association between exposure and outcome if it existed?	***NA***	***NA***	***NA***	***NA***	***NA***	***NA***	***NA***	***NA***	***NA***	***NA***	**N**	**N**	**Y**	**Y**	**Y**
8. For exposures that can vary in amount or level, did the study examine different levels of the exposure as related to the outcome (e.g., categories of exposure, or exposure measured as continuous variable)?	**Y**	**Y**	**N**	**Y**	**N**	***NA***	**Y**	***NA***	**Y**	**Y**	**Y**	**Y**	**Y**	***NA***	**Y**
9. Were the exposure measures (independent variables) clearly defined, valid, reliable, and implemented consistently across all study participants?	**Y**	**Y**	**Y**	**Y**	**N**	**Y**	**Y**	**Y**	**Y**	**Y**	**Y**	**Y**	**Y**	**Y**	**Y**
10. Was the exposure(s) assessed more than once over time?	**N**	**N**	**N**	**N**	**N**	**N**	**N**	**N**	**N**	**N**	**N**	**N**	**N**	**N**	**Y**
11. Were the outcome measures (dependent variables) clearly defined, valid, reliable, and implemented consistently across all study participants?	**Y**	**Y**	**Y**	**Y**	**Y**	**Y**	**N**	**N**	**Y**	**Y**	**N**	**N**	**N**	**Y**	**N**
12. Were the outcome assessors blinded to the exposure status of participants?	***NA***	***NA***	***NA***	***NA***	***NA***	***NA***	***NA***	***NA***	***NA***	***NA***	***NA***	***NA***	***NA***	***NA***	***NA***
13. Was loss to follow-up after baseline 20% or less?	***NA***	***NA***	***NA***	***NA***	***NA***	***NA***	***NA***	***NA***	***NA***	***NA***	**Y**	**Y**	**N**	**Y**	**Y**
14. Were key potential confounding variables measured and adjusted statistically for their impact on the relationship between exposure(s) and outcome(s)?	**N**	**N**	**N**	**N**	**N**	**N**	**N**	**N**	**N**	**N**	**N**	**N**	**N**	**N**	**N**
Rating overall	Good	Good	Fair	Fair	Poor	Fair	Fair	Poor	Fair	Fair	Fair	Fair	Fair	Good	Good

1 = Abdo et al. (2019) [36], 2 = Alduraidi et al. (2017) [37], 3 = Crea et al. (2015) [38], 4 = Georgiadou et al. (2020) [39], 5 = Horta et al. (2019) [40], 6 = Redko et al. (2015) [41], 7 = Carlsson, Mortensen et al. (2006) [42], 8 = Huijts et al. (2012) [43], 9 = Opaas & Varvin (2015) [44], 10 = Teodorescu et al. (2012) [45], 11 = Carlsson et al. (2005) [46], 12 = Carlsson et al. (2010) [47], 13 = Carlsson, Olsen et al. (2006) [48], 14 = Kinzie et al. (2012) [49], 15 = Opaas et al. (2016) [50]

*Y Yes, N No, NA Not Applicable, CD Cannot Determine, NR Not Reported*

## Discussion

The aim of this literature review was to highlight the evidence on the HRQOL of refugees, gain awareness for their needs, as well as present the elements having a greater impact on it.

In the studies selected, we can notice a wide heterogeneity among the scores obtained, in particular when referring to the general population of refugees: from a minimum score of 38.0 for the environmental domain, to a score of 73.1 for the physical domain. When observing the clinical group of refugees, lower outcomes were assessed for the physical and the psychological domain, scoring also below 30.0, while higher scores were assessed for the environmental domain reaching a value of 50.0. Nevertheless, it is possible to outline some elements having a considerable repercussion on specific domains and the differences among the two groups of refugees, general and clinic population, that we defined.

Regarding the Physical health domain, lower scores could be related to difficulties to access and understanding of a new healthcare system which might be demanding in terms of documents needed to access and cost of services. In particular, the lack and/or misuse of interpreters and culturally sensitive approach, which undermine the delivering of a correct and efficient treatment, represents one of the most decisive barriers to access healthcare service especially for refugees and asylum seekers [74–78]. However, we can notice that for this domain are observed the highest score for the general population of refugees and one of the lowest for the clinical group. The reason can be deemed by a high level of somatization of mental disorders [79–81], or the effect of past severe traumas, among which torture, that can be the cause of current physical pain for clinical refugees [82] and an “healthy migrant effect” characterizing individuals belonging to the general population [83].

Low scores in the Psychological health domain, that characterize particularly the population of clinical refugees, are bound to the traumatic and stressful events experienced which can have direct impact on psychological health [84]. Moreover, the outcomes of this domain could be also worsened by a lack of trained mental practitioners and/or language interpreters to deliver culturally sensitive treatment that is needed to disclose psychological symptoms. This paucity can worsen individual suffering by increasing integration obstacles and difficulties to access the health care system. There is limited but consistent evidence regarding the positive impact of interpreters in the quality of healthcare delivered, as capable to address the challenges related to specific social and cultural context. In particular, it was found to have a positive effect on utilization of preventive screening services, a decrease of the economic burden related to overtesting and unnecessary treatment, as well as a higher rate of hospitalization. In general, the use of trained interpreters leads to a more efficient health process, better outcomes and satisfaction of the patients [85] leading to higher scores in the Psychological health domain as well as in the Physical one. Another critical element undermining this outcome is the spread stigmatization of mental disorders, a factor that is deeply felt and prevents refugees from receiving psychiatric care [86]. Moreover, notice that the majority of the studies included in the general population group have been performed in UMIC, i.e., countries where access to healthcare and especially to mental healthcare is very poor or non-existent. This might contribute lowering the scores that we currently observe for the general population.

The Social relationship domain is strongly bound to community loss and cultural gap experienced in the new country. Family separation is an element that negatively affects the HRQOL in the Social relationship as well as in the Psychological domain due to a lack of supportive network, causing emotional distress and affecting their integration process [87]. This interpretation is supported by the outcomes observed in the study of Georgiadou et al. [53], in which there were observed major gaps between the two groups of married refugees separated by their partners and not, especially in the score of the Social relationship domain. The individuals included in the clinical population of refugees, are characterized by major traumatic events that can have a significant impact on social relationships. Indeed, in case of people suffering from PTSD a common behavior is the avoidance of interpersonal triggers which leads to distancing and detachment [88]. PTSD does also have a negative impact on intimacy and sexuality [89], therefore having a direct impact on the score of this domain. When referring to other mental disorders, the literature support the hypothesis that larger social networks represent a protective factor against depression and anxiety [90, 91]. However it is hard to untangle the causal influence of one to the other. Moreover, in some studies, it was hard to get answers regarding the question on sex life satisfaction so the score was not provided at all [52, 63].

Regarding the Environment domain, we observe a misalignment between the two groups. For the group of general population refugees, it represents the domain with the lowest scores as for the group of clinical population of refugees it is, almost for all the studies included, the highest. The reason for having lower scores in the environmental domain for the group of the general population of refugees could be bound to the low level of employment among refugees [92], which can directly affect the outcomes of this domain concerning financial stability, living space condition, the opportunity for leisure activities, quality of healthcare services and transportation. For example, in Jordan, the majority of Syrian refugees rely on humanitarian assistance as the unemployment rate is 77.8, and 82% of them live below the poverty line [93]. More positive values are shown regarding the sample of Palestinian refugees in Jordan in the paper by Alduraidi et al. [51], having an unemployment rate of 42.9 and 29.7% of them living below the poverty level. These values can be tied to the fact that the majority of Palestinian refugees in Jordan have full Jordanian citizenship, an element that can allow to major integration and benefits, relevant in determining higher scores in all domains. Unemployment creates a chain effect leading people to be incapable to satisfy primary needs, forcing children to be removed from school and a lack of access to healthcare services. There is strong evidence that economic and social factors, such as education [94], access to healthcare and social exclusion [95], as well as unemployment itself [96], are determinants of the health status. For this reason, a notably lower value of HRQOL in the environmental domain might be the direct reflection of those unfavourable elements. Those elements might also be fundamental in determining the outcome for the psychological domain, as a higher recognition of social support leads to a greater sense of control of environmental changes and recognition of personal identity [97]. When referring to the clinical refugee group, unemployment and its related issues might be overshadowed by severe mental distress experienced and by a more socially generous welfare state, so a major assistance in terms of financial benefits, that might characterize the high-income countries in which all those studies were performed. Moreover, the direction of causality is complex to determine as a mental disorder may result in social isolation and unemployment [98].

In the literature, clear cutoff values for the WHOQOL-Bref have not been set yet. Skevington et al. [47] refer to a cutoff of 50 to make a distinction between good and bad HRQOL, whereas other studies set a cutoff of < 60 to be an appropriate indicator for older adults with poor HRQOL [99, 100]. The studies selected for this review are not considering samples of older adults so we decided to not to rely on this particular cutoff.

To have a clearer perspective on the values shown by the selected studies in this review, we can consider the results obtained by Skevington et al. [47], who recruited 11,830 adults in 23 countries from the general population with a mean age of 45. Almost all the studies that have been included in this review scored lower than the average WHOQOL-Bref scores in Skevington et al.’s study, all above 50 and therefore in the area defined as “good” HRQoL by the same authors. There are two exceptions. Georgiadou et al. [53], considering just married individuals, reported HRQOL consistently higher in all the domains, and Alduraidi et al. [51], slightly lower for the physical and the social domain.

One of the main limitations of this review concerns the selection of participants in respect to their language skills: in some studies not knowing the language of the hosting country was an element of exclusion [57, 59], so refugees who might have major struggles of integration or recently arrived were not included in the sample. In other cases people were selected among native speakers of a certain language in which the questionnaires were translated into. Moreover, selection bias might be a problem more specifically when considering patients in the samples of studies included in the clinical refugee group because of the exclusion of patients not ill enough to be admitted to certain psychiatric facilities or, on the other hand, having a too serious medical and neurological illness, being under current suicidal risk, having active psychotic episodes or drug addiction. Those criteria lead to a non-random exclusion of individuals making it harder to draw general conclusions on the population of refugees and misrepresentation of the ones having a mental disorder. Another issue is related to the lack of information regarding whether an individual is receiving some kind of treatment during the study period [59] and more often if those had received treatment in the period before the study started. This information and the one regarding how long refugees have been settled into the community of the hosting country are fundamental to draw a clear baseline for this group and have a distinct view on which elements impact their HRQOL. Moreover, lots of different significant events can affect the outcomes that, whether positive (receiving citizenship, finding a job, being reunited to the family or separate from a toxic relationship) or negative (serious illness, losing a job), should be taken into account for inferences.

## Conclusions

This is the first systematic literature review that includes exclusively studies with populations of refugees living in the community of the hosting country and which considers studies assessing the health-related quality of life using the same evaluation tool. Several factors can explain the outcomes observed in the population of interest, which, for both groups, are lower than the scores reported by Skevington et al.’ general population. Lower employment rate and income, loss of family and social network, lack of full access to healthcare services, higher rate of mental disorders are an example of those factors, which incidence is higher in refugees populations. Among the two groups different patterns could be outlined considering each domain of HRQOL: higher scores for the Physical and lower for the Environment domain when considering the general population of refugees and higher scores for the Environment and lower for the Psychological domain when referring to the clinical one. Additionally, it is observed that individuals who were included in the clinical refugee group have a lower quality of life in respect to the general population of refugees. These lower scores are probably due to having a higher rate of mental distress and being more exposed to somatization, stigmatization and barriers to access the healthcare system of the hosting country in respect to the general population of refugees.

The overview presented should help to take awareness on the complexity of the experience of this population and acknowledge the multitude of elements determining their HRQOL. The WHOQOL-Bref appears to be a good tool for this purpose and could be used for future investigations as a tool to assess the effectiveness of integration policies in hosting countries.

## Supplementary Information

**Additional file 1.**

## Data Availability

Not applicable.

## Abbreviations

EQ-5D: EuroQoL-5 dimensions
HICs: High Income Countries
HRQOL: Health Related Quality of Life
HSCL-25: Hopkins Symptom Check List-25
HTQ-PTSD: Harvard Trauma Questionnaire-PTSD
PTSD: Post-traumatic Stress Disorder
QOL: Quality of Life
RCTs: Random Control Trials
SF-12: 12-item Short Form Health Questionnaire
SF-36: 36-item Short Form Health Questionnaire
UMICs: Upper-middle Income Countries
WHO: World Health Organization
WHO-5: World Health Organization - Five Well-Being Index
WHOQOL-BREF: World Health Organization Quality of Life Bref
