# Supplementary material for: Health-related quality of life of refugees: a systematic review of studies using the WHOQOL-Bref instrument in general and clinical refugee populations in the community setting
Source: Confl Health. 2021 Jun 2;15:44. doi: 10.1186/s13031-021-00378-1 (PMC8173726; doi:10.1186/s13031-021-00378-1)
Supplement: Supplementary file 1 — Additional file 1. [file 13031_2021_378_MOESM1_ESM.pdf]

C H A R A C T ER I S T I C S O F S T U D I ES

General Population of refugees

Abdo et al. (2019)	
Methods	**Study design:** comparative cross-sectional study
Purpose	To assess the QOL of Syrian refugees residing outside camps in Jordan and compare it to the QOL of Jordanians (having Low and Average Socioeconomic Status) and to other refugees and populations around the globe.
Participants	655 adults from Syria (208 LSES Jordanians, 712 ASES Jordanians)<br>**Diagnosis:** none<br>**Age:** 18-29 (17%), 30-39 (31.4%), 40-49 (32.7%), 50-59 (13.7%), > 60 (5.7%)<br>**Sex:** 40,6% men, 59,4% women<br>**Location:** Al-Husun, Jordan
Outcome	**Primary outcome:** WHOQOL-Bref<br>**Secondary outcome: -**
Baseline characteristics	87.4% unemployed, 89.4% married, 7% illiteracy, 39.8% free from disease
Adherence and completion	- Only answered 4.4% of the Syrians answered to the following item of the social relationships QOL: “How satisfied are you with your sex life?”<br>- Male less compliant (less representation of then male perspective)
Notes	**Date of the study:** Feb.-Aug 2017<br>**Funding source:** This project was funded by the Deanship of Research at Jordan University of Science and Technology, Grant Number 20170218. The funders had no role in study design, data collection and analysis, decision to publish, or preparation of the manuscript.<br>**Declaration of interest among primary researchers:** no declaration
Risk of bias	Attrition bias: missing data for the social relationship domain

Alduraidi et al. (2017)	
Methods	**Study design:** comparative cross-sectional study
Purpose	To compare HRQOL of Palestinian refugees in Jordan who live inside camps with those who live outside camps.
Participants	91 adults from Palestine living in the surrounding of Abu Nsair community (and 86 living in Baqa’s camp)<br>**Diagnosis:** none<br>**Age:** mean age 36<br>**Sex:** 48.4%% men, 51.6 % women<br>**Location:** Jordan
Outcome	**Primary outcome:** WHOQOL-Bref<br>**Secondary outcome: -**
Baseline characteristics	65.9% married, 42.9% unemployed, 70.3% living above the poverty level
Adherence and completion	10% of refugees refused to participate and no missing data.

Notes	**Date of the study:** Oct. - Nov. 2015<br>**Funding source:** none<br>**Declaration of interest among primary researchers:** no declaration
Bias	Selection bias: illiterate were excluded<br>Reporting bias: no study protocol available

Crea et al. (2015)	
Methods	**Study design:** comparative cross-sectional study
Purpose	To compare the perceived quality of life (QoL) among refugees living in urban and camp-based environments in sub-Saharan Africa, and to assess the influences of both the environment and the perceived environment on refugees' reported physical health.
Participants	n. adults in 334 from Eastern, Central and Northern/Western Africa (and 96 living in Camp)<br>**Diagnosis:** none<br>**Age:** -<br>**Sex:** 50.9% men, 44.9% women<br>**Location:** Johannesburg and Pretoria, South Africa
Outcome	**Primary outcome:** WHOQOL-Bref<br>**Secondary outcome: -**
Baseline characteristics	-
Adherence and completion	Small amount of missing data
Notes	**Date of the study:** June 2012- Aug. 2013<br>**Funding source:** not stated<br>**Declaration of interest among primary researchers:** no declaration
Bias	Attrition bias: Psychological Health domain and Social Relationships domain were removed a priori as considered to be sensitive to local cultural norms.

Georgiadou et al. (2020)	
Methods	**Study design:** comparative cross-sectional study
Purpose	Understand how separation from marital partner impact quality of life among Syrian refugees with a residence permit.
Participants	119 adults from Syria (93 Partner + and 26 Partner -)<br>**Diagnosis:** none<br>**Age:** mean age 38.83<br>**Sex:** 59.7% men, 40.3% women<br>**Location:** Erlangen, Germany
Outcome	**Primary outcome:** WHOQOL-Bref<br>**Secondary outcome:** Posttraumatic stress disorder (Essen Trauma Inventory), Depression (PGQ-9), Generalized anxiety disorder (GAD-7), Satisfaction of marriage, Sense of coherence (SOC-13), Social support (F-SozU)
Baseline characteristics	100% are married, 23.4 is duration in Germany in month, 9.8 years of education, 88.7 are parents.
Adherence and completion	The recruiting method was registry-based study with a response rate of 38.6%

Notes	**Date of the study:** July-Dec 2017<br>**Funding source:** ELAN Fund (16-12-21-1- Georgiadou) of the Medical Faculty of Friedrich Alexander University of Erlangen–Nuremberg.<br>**Declaration of interest among primary researchers:** no declaration
Bias	Reporting bias: no protocol available

Horta et al. (2019)	
Methods	**Study design:** cross-sectional study
Purpose	To measure their quality of life and to understand the needs and expectations of African refugee families in relation to health.
Participants	31 adults from Angola, Congo, Senegal and other.<br>**Diagnosis:** none<br>**Age:** -<br>**Sex:** 9.7% men, 90.3% women<br>**Location:** Brazil
Outcome	**Primary outcome:** WHOQOL-Bref<br>**Secondary outcome:** -
Baseline characteristics	Average months spent in Brazil 39.16 months
Adherence and completion	Small sample obtained through snowballing method
Notes	**Date of the study:** Aug 2016- April 2018<br>**Funding source:** not stated<br>**Declaration of interest among primary researchers:** no declaration
Bias	Reporting bias: no study protocol available

Redko et al. (2015)	
Methods	**Study design:** cross-sectional study
Purpose	Translation and validation of the WHOQOL-BREF for general use in Somali refugee populations.
Participants	303 adults from Somalia<br>**Diagnosis:** none<br>**Age:** mean age 46.5<br>**Sex:** 54.1% men, 42.6% women<br>**Location:** Usa
Outcome	**Primary outcome:** WHOQOL-Bref<br>**Secondary outcome: -**
Baseline characteristics	(survisiors, tortured, educ)
Adherence and completion	It was not possible to keep record of the number of potential respondents who refused to participate in this study. However Missing data were insignificant and no participants were completely excluded from analysis.
Notes	**Date of the study:** Sept. 2012- June 2013<br>**Funding source:** Wright State University Boonshoft School of Medicine Emerging Science Seed Grant, Dayton, OH.<br>**Declaration of interest among primary researchers:** no declaration

Bias	Selection bias: due to hard-to-reach population (due to their refugee status and because they are extremely suspicious of the surrounding community they live in, including potential research participation.), the sample is not representative of the population.

Clinical Population of refugees

Carlsson, Mortensen et al. (2006)	
Methods	**Study design:** cross-sectional study
Purpose	To describe a group of tortured refugees referred to the Rehabilitation and Research Centre for Torture Victims (RCT) and to study the importance of past trauma/torture and post-migratory factors for the present symptoms of PTSD, depression, anxiety and for health-related quality of life.
Participants	63 adults from Iraq, Afghanistan, Iran and other (not specified)<br>**Diagnosis:** PTSD, anxiety, depression<br>**Age:** mean age 37.8<br>**Sex:** 100 % men<br>**Location:** Copenhagen, Rehabilitation and Research Centre for Torture Victims (RCT), Denmark
Intervention	Multidisciplinary treatment (a combination of psychotherapy, physiotherapy/medical help, and social assistance).
Outcome	**Primary outcome:** WHOQOL-Bref, Anxiety and depression (HSCL-25, HDS), PTSD (HTQ)<br>**Secondary outcome:-**
Baseline characteristics	All participants were exposed to torture
Adherence and completion	missing values <63 due to factors such as participants' problems in remembering, concentrating or sharing certain data.
Notes	**Date of the study:** Jan 2001- 15 May 2002<br>**Funding source:** supported by Aase og Ejnar Danielsens Fond, Elly Valborg og Niels Mikkelsens Fond, FLS Industries Gavefond, Henrik Henriksens Fond, Illum Fondet, Læge Sofus Emil Friis.<br>**Declaration of interest among primary researchers:** no declaration
Bias	Reporting bias: no protocol available<br>Detection bias: questionnaires translated orally when needed (so not standardized).

Huijts et al. (2012)	
Methods	**Study design:** cross-sectional study
Purpose	To investigated the relationship between coping style, posttraumatic stress disorder (PTSD) symptoms, and quality of life in traumatized refugees.
Participants	335 adults from different countries of origin<br>**Diagnosis:** PTSD<br>**Age:** mean age 41.9<br>**Sex:** 74.9% men, 25.1% women<br>**Location:** Foundation Centrum '45, Oegstgeest, The Netherlands
Outcome	**Primary outcome:** WHOQOL-Bref, Coping style (COPE-EASY-32), PTSD (HTQ)<br>**Secondary outcome: -**
Baseline characteristics	Information on the traumas experienced
Adherence and completion	Full information modeling was used to deal with missing values and all models were estimated using the maximum likelihood estimator (ML).

Notes	**Date of the study:** 2003- 2010<br>**Funding source:** not stated<br>**Declaration of interest among primary researchers:** no declaration
Bias	Selection bias: Be sufficiently fluent in Dutch to complete research procedures.<br>Other bias: Use of invalidated translations of WHOQOL-Bref.

Opaas & Varvin (2015)	
Methods	**Study design:** cross-sectional study
Purpose	The aim was to investigate the early and cumulative exposure of participants to adversities or PTEs (Pre-Flight experiences) preceding flight, and to analyze these in relation to the participants' mental health and QOL.
Participants	54 adults from Middle Eastern countries<br>**Diagnosis:** PTSD, anxiety, depression<br>**Age:** mean age 39.3<br>**Sex:** 64.8% men, 35.2% women<br>**Location:** Two general mental health outpatient clinics and six psychotherapists in publicly funded individual practices, Oslo (Norway)
Outcome	**Primary outcome:** WHOQOL-Bref, Adverse and potentially traumatic childhood experiences (PTCEs), assessment of PTSD (HTQ), anxiety and depression (HSCL-25)<br>**Secondary outcome: -**
Baseline characteristics	Unemployment 77.8%
Adherence and completion	Among the 72 patients asked to participate, 18 patients declined
Notes	**Date of the study:** 2006-2009<br>**Funding source:** the Norwegian Centre for Violence and Traumatic Stress Studies.<br>**Declaration of interest among primary researchers:** no declaration
Bias	Attrition bias: 25% patients didn't accept to be included in the study (not wanting to share in detail their traumas, lack of trust in interpreters

Teodorescu et al. (2012)	
Methods	**Study design:** cross-sectional study
Purpose	To assess the presence and level of posttraumatic growth, posttraumatic stress symptoms, depressive symptoms, post-migration stressors and quality of life, and their relationship in a population of multi- traumatized refugees years after resettlement in Norway. To test a model for predicting quality of life among multi-traumatized refugees.
Participants	55 adults from different countries<br>**Diagnosis:** PTSD, anxiety, depression<br>**Age:** mean age 42<br>**Sex:** 58% men, 42% women<br>**Location:** Outpatient departments of four hospitals from South- Eastern Norway
Outcome	**Primary outcome:** WHOQOL-Bref, Life events Checklist (LEC), Clinician Administered PTSD Scale (CAPS) and Structural Clinical Interview for DSM-IV-TR PTSD module(SCID-PTSD), International Neuropsychiatric Interview 5.0.0 (MINI), Impact of Event Scale- Revised (IES-R), Posttraumatic Growth Inventory Short Form (PTGI-SF) and HSCL-25 depression scale.<br>**Secondary outcome:** -

Baseline characteristics	Number and type of traumas were reported, 83.3% of participants have been residing in Norway for a period longer than >10
Adherence and completion	Two subjects rescinded consent and six patients failed to return the questionnaires or to complete several scales and were thus excluded.
Notes	**Date of the study:** 1 Nov 2008 - Nov 2009<br>**Funding source:** not stated<br>**Declaration of interest among primary researchers:** no declaration
Bias	Selection bias: Patients were excluded if they suffered from any serious medical or neurological illness or organic mental disorder, were strongly sedated under current medication, had an active psychotic episode, were currently considered a high suicide risk or had insufficient knowledge of the Norwegian language.<br>Detection bias: It was not inquired whether or not patients received language help when completing the questionnaires at home.

Carlsson et al. (2005)	
Methods	**Study design:** longitudinal study
Purpose	The aim of this study was to examine changes in symptoms of PTSD, depression, and anxiety, and in health-related quality of life during treatment in traumatized refugees.
Participants	55 adults from Iraq, Iran and Afghanistan<br>**Diagnosis:** PTSD, anxiety, depression<br>**Age: -**<br>**Sex:** 72.7% men, 27.3% women<br>**Location:** Copenhagen, Rehabilitation and Research Centre for Torture Victims (RCT), Denmark
Intervention	Multidisciplinary treatment (a combination of psychotherapy, physiotherapy/medical help, and social assistance).
Outcome	**Primary outcome:** WHOQOL-Bref, Anxiety and depression (HSCL-25, HDS), PTSD (HTQ)<br>**Secondary outcome:** -
Baseline characteristics	Available information regarding the traumas experienced
Adherence and completion	69 agreed to participate, 7 of those dropped the follow up (but didn't differ from the rest of the sample) and overall 55 patient had complete baseline and follow-up data
Notes	**Date of the study:** 2001-2002<br>**Funding source:** Aase og Ejnar Danielsens Fond, Elly Valborg og Niels Mikkelsens Fond, FLS Industries Gavefond, Henrik Henriksens Fond, Illum Fondet, Læge Sofus Emil Friis.<br>**Declaration of interest among primary researchers:** no declaration
Bias	Detection bias: questionnaires translated orally when needed (so not standardized).

Carlsson et al. (2010)	
Methods	**Study design:** longitudinal study
Purpose	The aim of this study was to examine long-term changes in symptoms of posttraumatic stress disorder, depression, anxiety, and in health-related quality of life in traumatized refugees 23 months after admission to multidisciplinary treatment.

Participants	45 adults from Iraq, Iran and Afghanistan<br>**Diagnosis:** PTSD, anxiety, depression<br>**Age:** mean age 39.2<br>**Sex:** 66.7% men, 33.3% women<br>**Location:** Copenhagen, Rehabilitation and Research Centre for Torture Victims (RCT), Denmark
Intervention	Multidisciplinary treatment (a combination of psychotherapy, physiotherapy/medical help, and social assistance).
Outcome	**Primary outcome:** WHOQOL-Bref, Anxiety and depression (HSCL-25, HDS), PTSD (HTQ)<br>**Secondary outcome:** -
Baseline characteristics	Information wether the patients have been subjected to Torture or have been soldiers.
Adherence and completion	Of 69 treated patients who participated to the study, 62 participated to the 9-month follow-up and 56 to the 23-month follow-up However 45 completed all the assessment.
Notes	**Date of the study:** 1 Jan 2001- 15 May 2002<br>**Funding source:** by Aase og Ejnar Danielsens Fond, Elly Valborg og Niels Mikkelsens Fond, FLS Industries Gavefond, Henrik Henriksens Fond, Illum Fondet, and Læge Sofus Emil Friis.<br>**Declaration of interest among primary researchers:** no declaration
Bias	Detection bias: questionnaires translated orally when needed (so not standardized).

Carlsson, Olsen et al. (2006)	
Methods	**Study design:** longitudinal study
Purpose	The aim of the study was to identify predictors of mental symptoms (posttraumatic stress disorder, depression, and anxiety), and of health-related quality of life in refugees 10 years after referral to the Rehabilitation and Research Centre for Torture Victims, and to study changes in mental health over time.
Participants	139 adults from Iran, Iraq, Lebanon<br>**Diagnosis:** PTSD, anxiety, depression<br>**Age:** mean age 44.7<br>**Sex:** 90.6% men, 9.4% women<br>**Location:** Copenhagen, Rehabilitation and Research Centre for Torture Victims (RCT), Denmark
Intervention	Multidisciplinary treatment (a combination of psychotherapy, physiotherapy/medical help, and social assistance).
Outcome	**Primary outcome:** WHOQOL-Bref, Anxiety and depression (HSCL-25), PTSD (HTQ)<br>**Secondary outcome:** -
Baseline characteristics	Most frequent torture methods experienced are reported and length of imprisonment
Adherence and completion	4 persons had missing data. When it comes to the 10 years follow up there was 63% of compliance.
Notes	**Date of the study:**1991-1994<br>**Funding source:** by Aase og Ejnar Danielsens Fond, Elly Valborg og Niels Mikkelsens Fond, FLS Industries Ga<br>**Declaration of interest among primary researchers:** no declaration
Bias	Detection bias: questionnaires translated orally when needed (so not standardized).

Kinzie et al. (2012)	
Methods	**Study design:** longitudinal study
Purpose	The purpose of this report is to describe the result of one-year treatment outcome of torture survivors.
Participants	22 adults from Iran, Afghanistan, Ethiopia and Somalia<br>**Diagnosis:** diverse<br>**Age:** mean age 48<br>**Sex:** 41% men, 59% women<br>**Location:** Refugee clinic, Oregon (USA)
Intervention	Standard psychiatric treatment with evaluation, diagnosis, appropriate medicine, supportive psychotherapy and counseling by ethnic counselors provided good outcomes.
Outcome	**Primary outcome:** WHOQOL-Bref, Disability and treatment effects (HTQ and Sheehan Disability Scale), PTSD (SPRIT test) and depression (CES-D)<br>**Secondary outcome: -**
Baseline characteristics	22 patients were torture. Diagnoses of these six patients not qualifying as torture victims included major depressive disorders (three), social phobia and attention deficit disorder (one), and schizophrenia (one).
Adherence and completion	3 refusals to participate, all from Somalia. Starting with 33 they ended up with 28 patients in the follow-up.
Notes	**Date of the study: Feb. 2009- Jan. 2010**<br>**Funding source:** Jacob & Valeria Langeloth Foundation, 2004 grant, for partial funding of this work.<br>**Declaration of interest among primary researchers:** no declaration
Bias	-

Opaas et al. (2016)	
Methods	**Study design:** longitudinal study
Purpose	This study's aims were to examine relationships of the RIM components with measures of anxiety, depression, posttraumatic stress, quality of life (QOL), employment, and exile language skills throughout 3 years.
Participants	51-49 adults from different countries, 56.9% from Middle East<br>**Diagnosis:** PTSD, anxiety, depression<br>**Age:** mean age 39.4<br>**Sex:** 64.7% men, 35.3% women<br>**Location:** Specialist mental health outpatient services with public funding, Norway
Intervention	All patients were offered psychotherapy, which to a certain extent addressed the patient's life history, traumatic experiences, current relationship issues, and problems in daily living. The therapies were of multiple theoretical orientations, not manualized, and not part of any special program for refugees.
Outcome	**Primary outcome:** WHOQOL-Bref, Anxiety and depression (HSCL-25), PTSD (HTQ)<br>**Secondary outcome: -**
Baseline characteristics	Formal PTSD Diagnosis, mean stay in Norway 11.2 years, mostly having Norwegian citizenship, mean education in their country of origin was 9.7 years.

Adherence and completion	90.2% of the original sample completed the study
Notes	**Date of the study: 2006-2009**<br>**Funding source:** by the Norwegian Centre for Violence and Traumatic Stress Studies, Oslo, Norway<br>**Declaration of interest among primary researchers:** no declaration
Bias	Detection bias: questionnaires translated orally when needed (so not standardized).
